# Editorial: Implementation of novel drugs and modern radiotherapy in the treatment of lymphoma patients

**DOI:** 10.3389/fonc.2023.1273598

**Published:** 2023-08-17

**Authors:** Mario Levis, Simone Ferrero, Annalisa Chiappella, Benedetto Bruno, Umberto Ricardi

**Affiliations:** ^1^Department of Oncology, University of Torino, Torino, Italy; ^2^Department of Molecular Biotechnology and Health Sciences, Hematology Division, University of Torino, Torino, Italy; ^3^Division of Hematology and Stem Cell Transplantation, Fondazione IRCCS Istituto Nazionale dei Tumori, Milano, Italy

**Keywords:** lymphoma, novel drugs, modern RT, radiotherapy, immunotherapy, microenvironment

Modern drugs, such as immunotherapy and biological agents, in combination with cell-based treatments are rapidly revolutionizing the treatment landscape of lymphoproliferative disorders (1). Alongside with the development of systemic regimens, radiotherapy have dramatically evolved through the reduction in treatment fields and doses and the continuous advancements in the precision of the delivery techniques (2, 3). These evolutions are expected to significantly improve the therapeutic index of lymphoma patients in several settings, and in particular to reduce treatment-related toxicity events (4). In addition, the synergistic association of novel systemic agents and modern radiotherapy needs to be explored for the promising “pro-immunogenic” role of RT in combination with immunotherapy agents in different hematological scenarios.

The aim of our Research Topic is to discuss the advances in multimodal treatment in lymphoma patients, with a main focus on: 1) feasibility, safety and biological rational of novel drugs along with their actual limitations and possible side effects; 2) robustness of modern radiotherapy in improving the quality of treatment; 3) biological and clinical rational of an integrated approach of systemic agents with modern radiotherapy techniques in lymphoma patients.

In the first paper, Margiotta-Casaluci et al. published a retrospective study from the Fondazione Italiana Linfomi (FIL), aiming to compare the outcomes and toxicity of rituximab, cyclophosphamide, doxorubicin and prednisone (R-CHOP) versus the more recent combination of bendamustine plus rituximab (R-B) as frontline therapy for grade 3A follicular lymphomas. The authors found a similar outcome and toxicity profile among the two treatment regimens after adjustment for age. However, patients older than 75 years were more likely to receive R-B than R-CHOP (16% vs 3%), in reason of a more favourable toxicity profile in unfit and frail patients.

The frailty of elderly patients is also the main topic of the review paper by Arcari et al.. In particular, the authors focused on the new treatment options for elderly patients with relapsed/refractory diffuse large B cell lymphoma not eligible to transplant or to chimeric antigen receptor (CAR) T-cell infusion. This paper offers a detailed overview on the standard treatment options and on the variety of emerging bispecific antibodies that have recently broadened the treatment landscape in this setting.

The third paper, by Ribeiro et al., is an original research, investigating the effectiveness of combined immunotherapy drugs *in-vitro* and *in-vivo* in animals against Burkitt lymphoma cells. The authors observed a remarkable and durable antitumor effect of a triplet-therapy composed by a bispecific antibody selectively targeting CD47 and CD19+ B-cells, a new generation anti-CD20 antibody and a phosphoinositide 3-kinase isoform δ (PI3Kδi) inhibitor. Moreover, the authors noticed that the upregulation of a particular gene, “so-called” GPR183, enhances the efficacy of that triplet combination.

The central role of immunotherapy in the actual treatment landscape of lymphoma patients is confirmed by the paper that Cellini et al. have published in this Research Topic. This review paper focused on the complex biological architecture of the tumor micro-environment surrounding and nurtured by Hodgkin’s lymphoma (HL) cells and on all the factors contributing to the immune evasion of HL. Alongside, the authors discuss in detail the success obtained by checkpoint inhibitors (CPI) in treating HL as single agents and as part of combination strategies.

The study by Oertel et al. is a secondary analysis of the German Hodgkin Study Group (GHSG) HD17 trial (5) that points out the importance of standardized quality assurance (QA) protocols to guarantee high quality RT processes. This study is innovative as it focuses on a topic with few literature data (6) such as the QA analyses of patients treated to limited target volumes in respect of the modern principles of involved-node RT and involved-site RT. In this study, the authors performed a detailed dosimetric analysis to retrospectively investigate for the impact of disease extension and localization on RT dose exposure to different organs at risk, with a particular focus on mediastinal organs. This study represents a first step toward future tailored RT-strategies, based on individualized risk-benefit assessments.

Finally, the study by Dabaja and Spiotto is a comprehensive overview on the changing role of RT for hematological malignancies from direct cell killing to immune cell priming. This includes a description on recent advancements in immunotherapy and adoptive cell therapy and, more in detail, on the pro-immunogenic role of RT in combination with monoclonal antibodies and other immunostimulatory agents. Furthermore, the authors discuss in detail the emerging role of RT as “bridging” and “priming” that facilitates CAR T cells engraftment and activity (7). This review paper presents the novel and future solutions to integrate RT in various combinations with systemic agents to offer the most effective and least toxic treatment for hematological malignancies.

In conclusion, this Research Topic provides insights into the synergistic implementation of novel drugs and modern RT techniques to treat lymphoma patients. Ongoing projects and future research will broaden our knowledge on the safety and effectiveness of innovative systemic agents and better clarify the role of multimodal integrated approaches with modern RT.

## Author contributions

ML: Writing – original draft, Writing – review & editing. SF: Writing – original draft, Writing – review & editing. AC: Writing – original draft, Writing – review & editing. BB: Writing – original draft, Writing – review & editing. UR: Writing – original draft, Writing – review & editing.

## References

[1] Chiappella A Santambrogio E Castellino A Nicolosi M Vitolo U. Integrating novel drugs to chemoimmunotherapy in diffuse large B-cell lymphoma. Expert Rev Hematol (2017) 10(8):697–705. doi: 10.100/17474086.2017.1350164 28665232

[2] Filippi AR Levis M Parikh R Hoppe B. Optimal Therapy for early-stage Hodgkin’s lymphoma: risk adapting, response adapting, and role of radiotherapy. Curr Oncol Rep (2017) 19:34. doi: 10.1007/s11912-017-0592-7 28365830

[3] Wirth A Mikhaeel NG Aleman BMP Pinnix CC Constine LS Ricardi U. Involved site radiation therapy in adult lymphomas: an overview of International Lymphoma Radiation Oncology Group guidelines. Int J Radiat Oncol Biol Phys (2020) 107:909–33. doi: 10.1016/j.ijrobp.2020.03.019 32272184

[4] Maraldo MV Levis M Andreis A Armenian S Bates J Brady J. An integrated approach to cardioprotection in lymphomas. Lancet Haematol (2022) 9:e445–54. doi: 10.1016/S2352-3026(22)00082-5 35512725

[5] Borchmann P Plütschow A Kobe C Greil R Meissner J Topp MS. PET-guided omission of radiotherapy in early-stage unfavourable Hodgkin lymphoma (GHSG HD17): a multicentre, open-label, randomized, phase 3 trial. Lancet Oncol (2021) 21:223–34. doi: 10.1016/S1470-20145(20)30601-X 33539742

[6] Aleman BPM Ricardi U van der Maazen RWM Meijnders P Beijert M Boros A. A quality control study on involved node radiation therapy in the European Organisation for Research and Treatment of Cancer/Lymphoma Study Association/Fondazione Italiana Linfomi H10 trial on stages I and II Hodgkin lymphoma: lessons learned. Int J Radiat Oncol Biol Phys (2023) S0360-3016(23):00455–8. doi: 10.1016/j.ijrobp.2023.05.011 37179034

[7] Fang PQ Gunther JR Wu SY Dabaja BS Nastoupil LJ Ahmed S. Radiation and CAR T-cell therapy in lymphoma: future frontiers and potential opportunities for synergy. Front Oncol (2021) 11:648655. doi: 10.3389/fonc.2021.648655 33842363 PMC8027336

